# Timed up-and-go performance is associated with objectively measured life space in patients 3 months after ischemic stroke: a cross-sectional observational study

**DOI:** 10.1007/s00415-022-11524-x

**Published:** 2022-12-22

**Authors:** Roland Rössler, Nikki Rommers, Eun-Kyeong Kim, Laura Iendra, Alexander Sofios, Eleftheria Giannouli, Erja Portegijs, Taina Rantanen, Denis Infanger, Stephanie Bridenbaugh, Stefan T. Engelter, Arno Schmidt-Trucksäss, Robert Weibel, Nils Peters, Timo Hinrichs

**Affiliations:** 1 Department of Sport, Exercise, and Health, University of Basel, Grosse Allee 6, 4052 Basel, Switzerland; 2 Basel Mobility Center, Department of Geriatric Medicine Felix Platter, Basel, Switzerland; 3 Department of Clinical Research, University Hospital Basel and University of Basel, Basel, Switzerland; 4 Department of Geography, University of Zurich, Zurich, Switzerland; 5 University Research Priority Program (URPP) Dynamics of Healthy Aging, University of Zurich, Zurich, Switzerland; 6 Department of Urban Development and Mobility, Luxembourg Institute of Socio-Economic Research (LISER), Esch-sur-Alzette, Luxembourg; 7 Department of Health Sciences & Technology, Institute of Human Movement Sciences and Sport, ETH Zurich, Zurich, Switzerland; 8 Neurology und Neurorehabilitation, University Department of Geriatric Medicine Felix Platter, Basel, Switzerland; 9 Department of Neurology & Stroke Center, University Hospital Basel, University of Basel, Basel, Switzerland; 10 Center for Human Movement Sciences, University Medical Center Groningen, University of Groningen, Groningen, the Netherlands; 11 Faculty of Sport and Health Sciences & Gerontology Research Center, University of Jyvaskyla, Jyvaskyla, Finland; 12 Neurology and Stroke Center, Klinik Hirslanden, Zurich, Switzerland

**Keywords:** Mobility limitation, Spatial behaviour, Quality of life, GPS, Mobility capacity

## Abstract

**Background:**

Stroke is a common cause of mobility limitation, including a reduction in life space. Life space is defined as the spatial extent in which a person moves within a specified period of time. We aimed to analyze patients’ objective and self-reported life space and clinical stroke characteristics.

**Methods:**

MOBITEC-Stroke is a prospective observational cohort study addressing poststroke mobility. This cross-sectional analysis refers to 3-month data. Life space was assessed by a portable tracking device (7 consecutive days) and by self-report (Life-Space Assessment; LSA). We analysed the timed up-and-go (TUG) test, stroke severity (National Institutes of Health Stroke Scale; NIHSS), and the level of functional outcome (modified Rankin Scale; mRS) in relation to participants’ objective (distance- and area-related life-space parameters) and self-reported (LSA) life space by multivariable linear regression analyses, adjusted for age, sex, and residential area.

**Results:**

We included 41 patients, mean age 70.7 (SD11.0) years, 29.3% female, NIHSS score 1.76 (SD1.68). We found a positive relationship between TUG performance and maximum distance from home (*p* = 0.006), convex hull area (i.e. area enclosing all Global Navigation Satellite System [GNSS] fixes, represented as a polygon linking the outermost points; *p* = 0.009), perimeter of the convex hull area (i.e. total length of the boundary of the convex hull area; *p* = 0.008), as well as the standard ellipse area (i.e. the two-dimensional ellipse containing approximately 63% of GNSS points; *p* = 0.023), in multivariable regression analyses.

**Conclusion:**

The TUG, an easily applicable bedside test, seems to be a useful indicator for patients’ life space 3 months poststroke and may be a clinically useful measure to document the motor rehabilitative process.

**Supplementary Information:**

The online version contains supplementary material available at 10.1007/s00415-022-11524-x.

## Background

Globally, stroke was the second-leading cause of death accounting for 11.6% [10.8–12.2%] of total deaths in 2019 [10]. If survived, stroke often results in permanent disability and limitations of mobility [16] which can be defined as “the ability to move oneself (either independently or by using assistive devices or transportation) within environments that expand from one’s home to the neighbourhood and to regions beyond” [46]. Many studies investigating mobility after stroke focused on physical function (e.g. strength, balance, walking speed) [3, 11, 14, 21, 24]. However, it appears more important whether a person can or cannot reach a certain location, irrespective of the form of transportation, as this might eventually affect the perceived quality of life [7, 34]. Therefore, life space seems to be a mobility outcome of high practical relevance as it describes the spatial behavior including the interaction between intrinsic capabilities of the person (e.g. walking ability, ability to climb stairs) and the demands of the extrinsic environment. Life space is defined as the spatial extent in which a person moves within a specified period of time [43]. Given the long-term consequences of a limited life space, regaining life space should be one of the main rehabilitation goals after stroke.

The University of Alabama at Birmingham Study of Aging Life-Space Assessment (LSA) is a widely used approach to evaluate older adults' life space due to its ease of use and high test–retest-reliability [2, 30]. Recently, it has been used in studies on patients with stroke [20, 23, 42, 47].

Life space can also be measured objectively using mobile devices utilizing global navigation satellite systems (GNSS; including e.g., GPS, GALILEO, GLONASS, BeiDou) [17, 18]. Specific tracking devices [4] or apps [28, 43] allow to continuously record the participants’ location and to analyze mobility parameters such as total distance or area covered over a specific period of time [43].

Despite the relevance for personal health and social interaction and the availability of methods and technology, it is only since very recently that researchers started to investigate life space after stroke. The existing literature describes positive associations of subjectively measured life space (i.e., questionnaire assessment) with physical function [20, 42, 47] and physical activity (i.e. step count) [23] in patients with stroke. The authors of those four studies highlight the need for further research on this topic [20, 23, 42, 47]. To the best of our knowledge, to date no study investigated objectively measured life space in patients with stroke using a tracking device. In a study including older adults with mild-to-moderate Alzheimer's disease, significant correlations between physical function as well as physical activity (i.e. steps per day) and life space (i.e. GPS-based area and perimeter measures) have been reported [43]. The current study aimed to describe the life space of patients three months after an ischemic stroke and to investigate the association between stroke severity, level of functional independence, as well as lower extremity physical function and life space.

## Methods

### Design

This study was part of the project “MOBITEC-Stroke” (“Recovery of mobility function and life-space mobility after ischemic stroke”; ISRCTN85999967) [38]. MOBITEC-Stroke is a prospective observational cohort study in which clinical evaluation and mobility measurements were performed at 3, 6, 9, and 12 months after ischemic stroke. At each visit, a 4-h battery of clinical assessments, functional tests, and questionnaires was performed. The GNSS measurement took place during 7 days following the appointment on site. Further details are described in the study protocol [38]. The present study is based on the analysis of the measurements at 3 months post stroke.

### Study population, inclusion criteria and exclusion criteria

The study targeted ambulatory patients after their first ischemic stroke living in their own homes.

The inclusion criteria were as follows: a first ischemic stroke within the past 3 months; being able to communicate verbally with the study personnel; understanding the study information and providing written informed consent. At least one of the following stroke-related symptoms potentially affecting gait and mobility had to be present: lower limb paresis or ataxia; stance/gait ataxia (cerebellar or sensory); visual disturbance/field defect; central vestibular deficit; attentional deficit/neglect. Participants had to be able to get up from a chair and sit down without external help and to walk for a minimum of 20 m at their own pace, with or without pauses, with or without a walking aid, but without the physical assistance of another person (self-report).

Potential participants were excluded if they were not living in their own home, were unable to walk without assistance (modified Rankin Scale, mRS > 3 points) [44]; had a severe cognitive impairment (Montreal Cognitive Assessment (MoCA) score < 21 or, < 20 for persons with ≤12 years of education) [13, 29], an acute psychiatric disorder (e.g. severe depression), or an advanced terminal illness. Further, an orthopaedic surgery of the lower extremities within the past year and ongoing rehabilitation measures following an inpatient surgical procedure at the time of stroke were exclusion criteria. Patients with major pre-stroke mobility limitations (major difficulties in walking or climbing stairs; self-report) were also not considered for the study.

All patients who had an acute ischemic stroke between October 2019 and March 2021 that presented at the Stroke Center, University Hospital Basel were screened for eligibility based on the above criteria and received standard-of-care rehabilitation based on the individual deficits at the Neurorehabilitation Center, Felix Platter. Eligible patients were contacted and offered to participate in the study. Details on sample size calculation (based on the longitudinal analyses of the primary outcome) have been reported elsewhere [38]. Recruitment was stopped once the targeted number of *N* = 59 participants was reached. We limited the current analyses to those participants with complete life-space data (i.e. *n* = 41).

### Questionnaire-based assessment of life space

We used the LSA questionnaire to assess life space. The LSA measures the extent of an individual’s movement within the past 4 weeks, categorized into 5 spatial levels, ranging from the participant’s bedroom to places outside the participants’ hometown, by self-report [2]. Besides the spatial extent of movement, it also assesses the frequency of travel to the 5 spatial levels and the need of assistance for that travel. The LSA takes into account the degree of independence, as well as in-house movement. It has shown a high test–retest-reliability [intraclass correlation of 0.96 (95% confidence interval 0.95–0.97)] [2, 30].

We used the German version of the LSA and calculated the composite score of the LSA summarising the attained life-space level, needed equipment or personal support and frequency of visits. The composite score ranges from 0 to 120 points with higher scores indicating better mobility [27].

### GNSS-based assessment of life space

Participants wore a small portable tracking device (uTrail, CDD Ltd., Athens, Greece) for 7 consecutive days. They received clear instructions (verbally and in print) on how to wear the device and charge the battery (overnight). We called the participants once during the observation period to check for potential issues. The device was designed to always stay on. It recorded GNSS data continuously, as long as it was not charging. Apart from the geographic locations (latitude and longitude) the device also logged the date and time as well as the velocity.

Missing or erratic data points (e.g., points with erratic timestamp) as well as extreme speed outliers (i.e., > 250 km/h) were excluded. For our analysis, we set the minimum GNSS recording duration of 6 h per day to ensure that enough hours of a day are recorded. Only study days with more than 6 h of GNSS recordings were defined as valid study days. To be a valid weekly session, a session had to contain at least one valid day. With these validation criteria, the number of “valid” weekly sessions was *n* = 41.

### Life-space metric calculation

Several life-space metrics were calculated for each valid weekly session. The metrics were first computed at a day level, and then, those daily metrics were aggregated to derive the weekly session of each participant.

### Selected life-space metrics

For each participant, following metrics were calculated:

Total distance travelled (km) is the sum of Euclidean distances between all the subsequent GNSS fixes projected in a planar coordinate system.

Maximum distance to home (km) is the maximum Euclidean distance between all GNSS fixes and home location in a planar coordinate system.

**Fig. 1:**
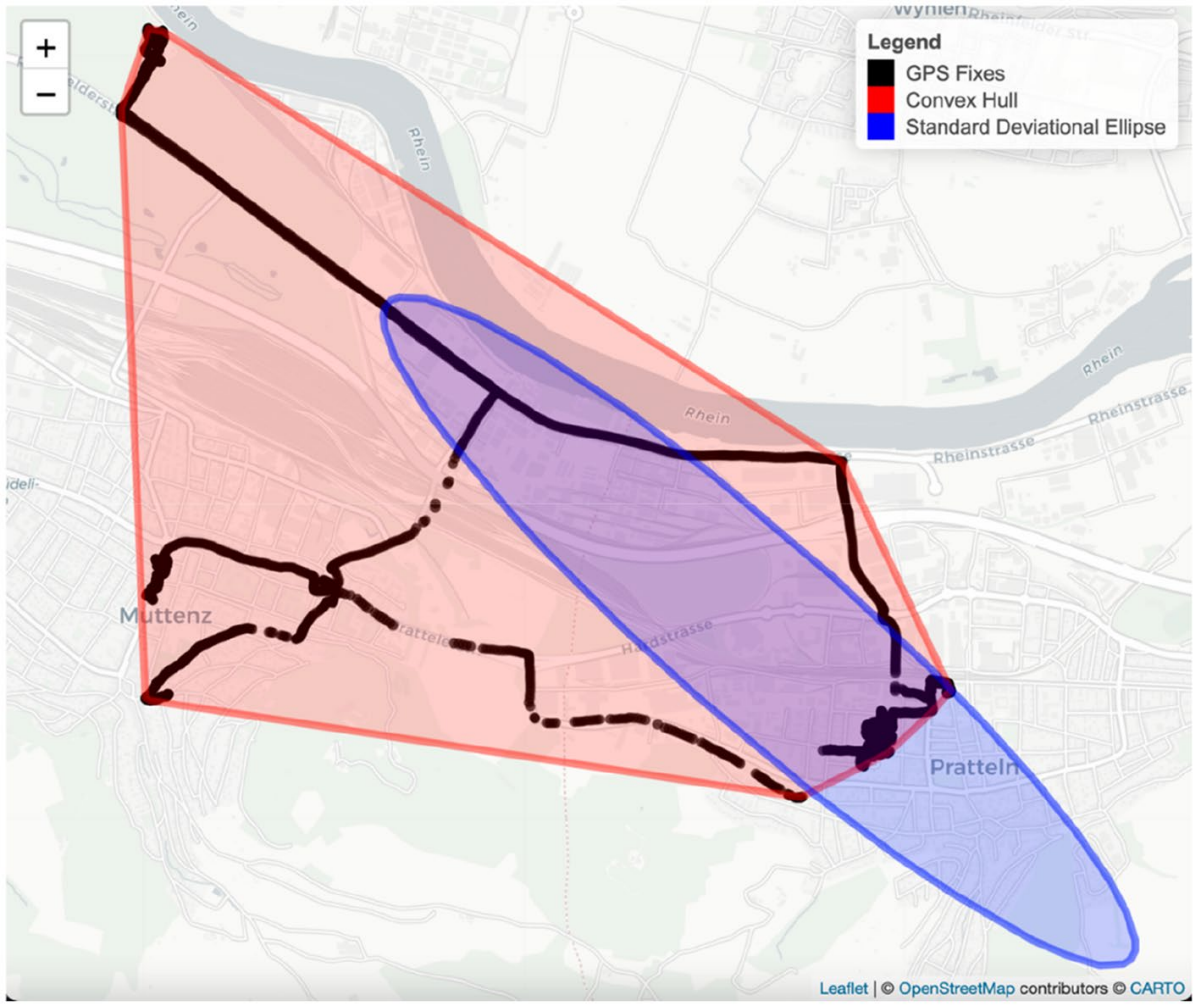
Life space of one day of an example participant with an area of the convex hull (red) and area of a standard deviational ellipse (blue) based on GNSS fixes

Area of the convex hull (km^2^) is defined as the smallest convex area enclosing all GNSS fixes and being represented as a polygon linking the outermost points with inner angles of less than 180 degrees (Fig. 1) [1].

The perimeter of the convex hull area (km) is defined as the total length of the boundary of the convex hull area.

Area of the standard deviational ellipse (km^2^) is defined by one standard deviation (SD) of GNSS point coordinates. The two-dimensional ellipse contains approximately 63% of GNSS points within its boundary [45] (Fig. 1).

### Physical function, functional independence and stroke severity

We used the timed up-and-go (TUG) test as an easily assessable, general measure of physical function. In the TUG, participants stand up from a chair, walk around a cone 3 m away and return to the chair to sit down again [25, 32]. Participants were allowed to use their walking aid and to use the armrest for support when getting up. The outcome of the TUG test was the total time in seconds from standing up (first visible movement) to sitting down (back of the participant touching the back of the chair). Participants were told to stand up, walk around the cone and sit back down on the chair as fast as possible while staying safe and performing the movements in a controlled manner.

Stroke severity (National Institutes of Health Stroke Scale; NIHSS [5]; 0–1 vs. ≥ 2) and level of functional independence (mRS [44]; 0–1 vs. ≥ 2) were assessed by a physician.

### Participant characteristics

Body height and body weight were measured by a trained assessor, body mass index was calculated. Via self-report, we assessed age, sex, residential area (i.e., urban/suburban/rural) [36], years of education (i.e., total number of school years and professional education), financial hardship [36], Instrumental Activities of Daily Living (IADL) [6], as well as comorbidities (Self-Administered Comorbidity Questionnaire; SCQ) [39, 41]. Further, we assessed cognitive function with the Montreal Cognitive Assessment (MoCA) [8]. Information on the type and location of the stroke (based on brain imaging) have been retrieved from the hospital records. We collected information about whether a private car is available or not.

### Statistical analyses

All participant characteristics and outcomes are described by the mean and standard deviation (SD) as well as median and range for numerical variables, and by frequency and percentage for count variables. We tested for potential differences regarding the participant characteristics between the participants with and without available GNSS data.

We visually inspected the distribution of the continuous variables using histograms to assess the distribution. All non-normally distributed variables were log-transformed. For each life-space-related dependent variable (i.e., LSA questionnaire, total covered distance per day, maximum distance from home, convex hull area, perimeter of the convex hull area, and the standard ellipse area), we fit univariable and multivariable linear regression models to examine the association between these outcomes and the following set of predictors (independent variables): dichotomized NIHSS score, dichotomized mRS, and the log-transformed TUG test result. Age, sex, and residential area were included as confounders.

To test for potential collinearity between predictors in the models, we calculated the variance inflation factor. We inspected distributions of the residuals using QQ-plots and evaluated homoscedasticity using residual plots. We report the unstandardized regression coefficients (B) with 95% confidence intervals (CIs) for each predictor to describe the association between the predictors and life-space outcome measures. The statistical analysis was carried out in R version 4.2.2 [33].

## Results

In total, we analyzed life-space data of *n* = 41 patients with stroke with an average age of 70.7 (SD 11.0) years. The sample included *n* = 12 females with a mean body height of 162.0 cm (SD 4.1) and a mean body mass of 67.5 kg (SD 13.4), and *n* = 29 males with a mean body height of 176.0 cm (SD 8.0) and a mean body mass of 80.0 kg (SD 14.3). On average, data collection on site took place 90.5 (SD 7.7) days (ranging from 74 to 110 days) post stroke. The weekly GNSS data collection started the day after the visit to the study site. Overall, the objectively measured life space showed a large variation from participants who barely left their home to participants who covered relatively large daily distances and areas.

Participant characteristics are described in Table 1 and a descriptive overview of the life-space data is provided in Table 2. There were no statistically significant differences (*p* > 0.14) in any of the demographic, clinical, and functional mobility outcomes assessed between the participants with available GNSS data (*n* = 41) and those for whom no valid GNSS data was available (*n* = 17).

**Table 1:** Characteristics of the study participants

Measure	Female	Male	Overall
	(*n* = 12)	(*n* = 29)	(*n* = 41)
BMI [kg/m2]			
Mean (SD)	25.8 (4.66)	27.6 (3.32)	27.0 (3.80)
Median [Min, Max]	25.2 [18.6, 33.2]	26.4 [22.0, 34.6]	26.4 [18.6, 34.6]
Timed up-and-go (TUG) [s]			
Mean (SD)	10.60 (3.67)	9.03 (2.90)	9.48 (3.18)
Median [Min, Max]	10.1 [5.70, 18.1]	8.57 [5.18, 17.9]	8.62 [5.18, 18.1]
mRS [0–6, lower is better]			
Mean (SD)	1.42 (0.52)	1.48 (0.83)	1.46 (0.75)
Median [Min, Max]	1 [1, 2]	1 [0, 3]	1 [0, 3]
NIHSS [0–42, lower is better]			
Mean (SD)	1.67 (1.92)	1.79 (1.61)	1.76 (1.68)
Median [Min, Max]	1.5 [0, 6]	1 [0, 6]	1 [0, 6]
School and education [years]			
Mean (SD)	11.5 (2.71)	13.8 (3.46)	13.1 (3.39)
Median [Min, Max]	11.5 [6.00, 15.0]	13.0 [8.00, 26.0]	13.0 [6.00, 26.0]
Financial situation			
No financial hardship	12 (100%)	25 (86.2%)	37 (90.2%)
Little restriction of social life	0 (0%)	3 (10.3%)	3 (7.3%)
Severe restriction of social life	0 (0%)	1 (3.4%)	1 (2.4%)
IADL [8–32, lower is better]			
Mean (SD)	11.9 (4.83)	11.3 (5.48)	11.5 (5.24)
Median [Min, Max]	11.0 [8.00, 22.0]	9.00 [8.00, 32.0]	9.00 [8.00, 32.0]
Leg paresis			
Yes	5 (41.7%)	17 (58.6%)	22 (53.7%)
No	7 (58.3%)	12 (41.4%)	19 (46.3%)
Stroke location			
Infratentorial stroke	4 (33.3%)	12 (41.4%)	16 (39.0%)
Middle cerebral artery stroke	6 (50.0%)	13 (44.8%)	19 (46.3%)
Other	2 (16.7%)	4 (13.8%)	6 (14.6%)
Comorbidities			
Heart disease	3 (25.0%)	9 (31.0%)	12 (29.3%)
High blood pressure	8 (66.7%)	18 (62.1%)	26 (63.4%)
Diabetes	0 (0%)	8 (27.6%)	8 (19.5%)
Depression	0 (0%)	2 (6.9%)	2 (4.9%)
Osteoarthritis, degenerative arthritis	2 (16.7%)	6 (20.7%)	8 (19.5%)
Availability of a private car	7 (58.3%)	17 (58.6%)	24 (58.5%)

*BMI* Body-Mass-Index, *TUG* Timed up-and-go test, *mRS* Modified Rankin Scale, *NIHSS* National Institutes of Health Stroke Scale, *IADL* Instrumental Activities of Daily Living

**Table 2:** Outcomes of subjective (LSA, questionnaire-based) and objective (GNSS-based) life-space assessments

Life-space outcome	Female	Male	Overall
	(*n* = 12)	(*n* = 29)	(*n* = 41)
LSA [0–120, higher is better]			
Mean (SD)	74.0 (23.8)	70.1 (28.3)	71.2 (26.8)
Median [Min, Max]	71.0 [38.0, 110.0]	72.0 [9.0, 120.0]	72.0 [9.0, 120.0]
Total covered distance [km]			
Mean (SD)	37.6 (24.2)	39.5 (31.5)	38.9 (29.3)
Median [Min, Max]	30.9 [0.5, 92.0]	34.8 [9.8, 177.0]	34.2 [0.5, 177.0]
Maximum distance from home [km]			
Mean (SD)	7.0 (7.6)	6.7 (11.4)	6.8 (10.4)
Median [Min, Max]	3.9 [0.1, 19.9]	2.6 [0.1, 56.2]	2.8 [0.1, 56.2]
Convex hull area [ km^2^]			
Mean (SD)	40.0 (72.6)	26.8 (67.8)	30.7 (68.6)
Median [Min, Max]	7.0 [0.0, 228.0]	2.8 [0.0, 283.0]	2.9 [0.0, 283.0]
Perimeter of the convex hull area [km]			
Mean (SD)	16.9 (18.3)	14.5 (22.7)	15.2 (21.3)
Median [Min, Max]	9.5 [0.2, 49.8]	6.7 [0.3, 119.0]	6.7 [0.2, 119.0]
Standard ellipse area [km^2^]			
Mean (SD)	20.1 (35.8)	17.6 (52.5)	18.3 (47.8)
Median [Min, Max]	4.9 [0.0, 120.0]	1.7 [0.0, 254.0]	1.9 [0.0, 254.0]

*LSA* University of Alabama at Birmingham Study of Aging Life-Space Assessment

**Fig. 2:**
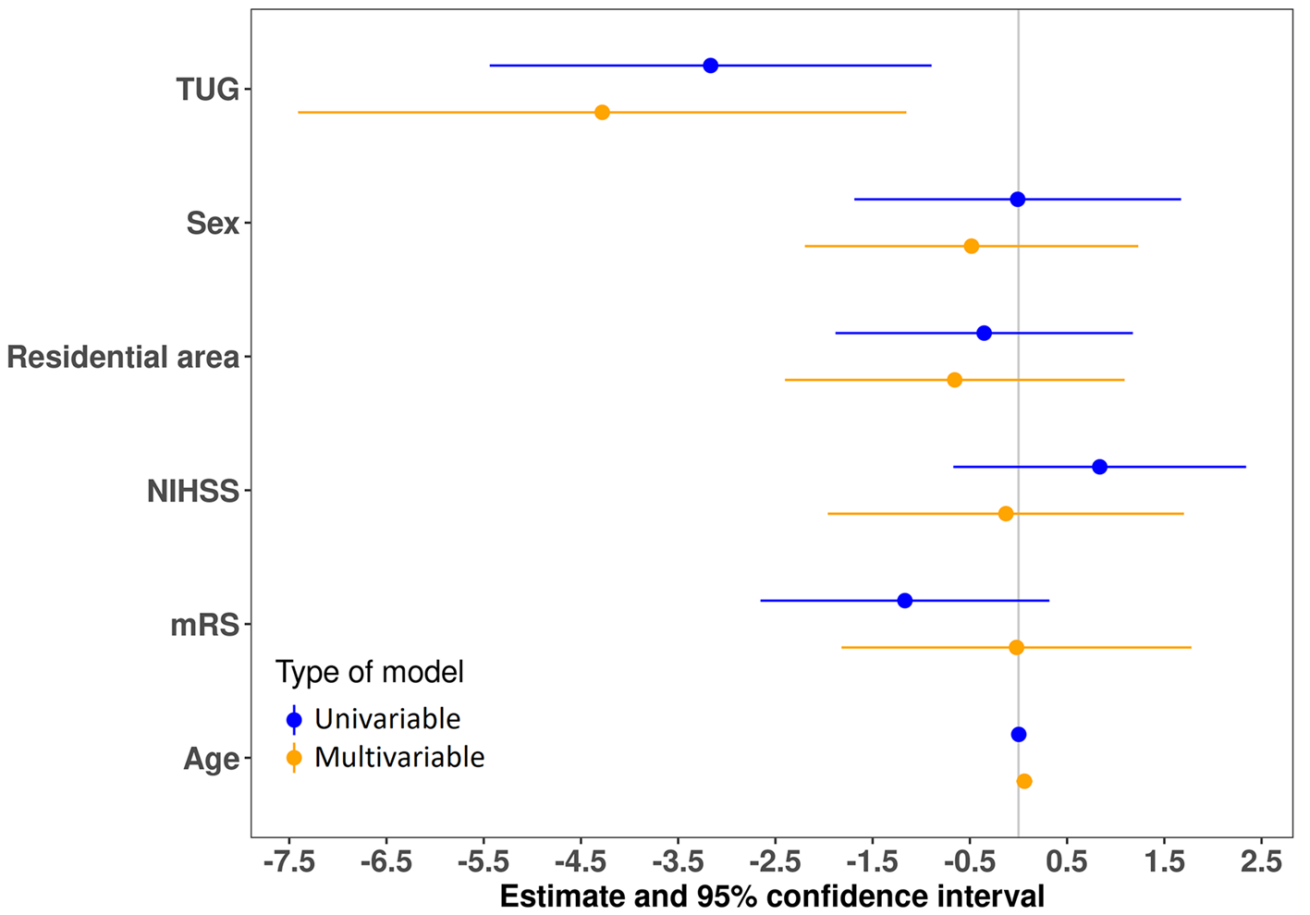
Visualization of the regression outcomes showing the unstandardized regression coefficients (B) and 95%-CIs of the models for the log-transformed convex hull area. The vertical line indicates the null value. *mRS* Modified Rankin Scale, *NIHSS* National Institutes of Health Stroke Scale, *TUG* Timed up-and-go test

**Fig. 3:**
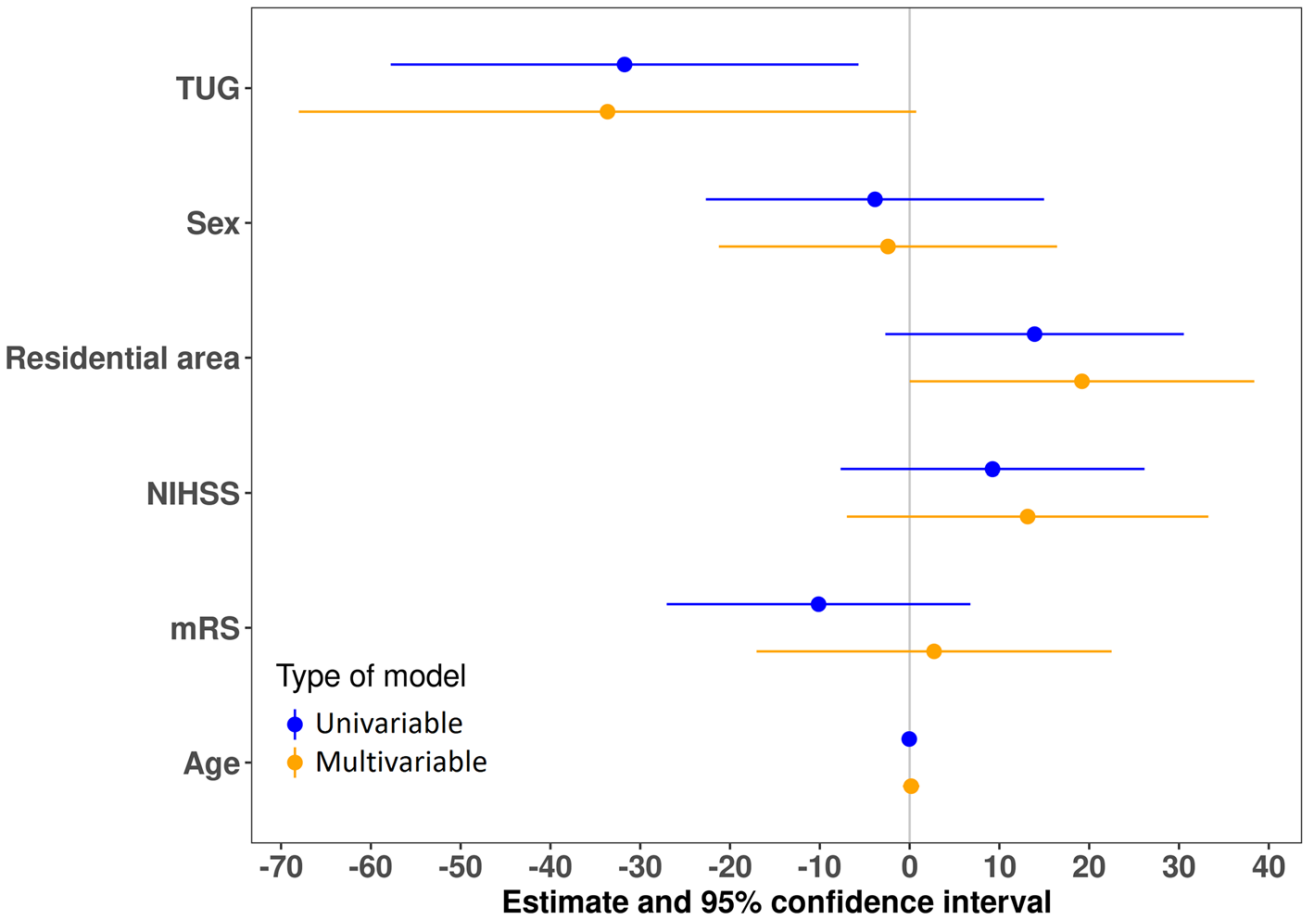
Visualization of the regression outcomes showing the unstandardized regression coefficients (B) and 95%-CIs of the models for the LSA score. The vertical line indicates the null value. *mRS* Modified Rankin Scale, *NIHSS* National Institutes of Health Stroke Scale, *TUG* Timed up-and-go test

The results of both univariable and multivariable regression models for the analysis of the convex hull area and the LSA score are visualized in Figs. 2 and 3, respectively. Further figures for the other analyses can be found in the online appendix.

In the following, we focus on the results of the univariable regression models. The TUG performance was positively associated (i.e., negatively associated with the TUG time) with the LSA questionnaire, maximum distance from home, convex hull area, perimeter of the convex hull area, and the standard ellipse area (with *p*-values ranging between 0.003 and 0.018). We did not find significant associations between any life-space measure and the NIHSS or mRS, with. The mRS, however, was borderline significantly associated (*p* = 0.053) with the standard ellipse area (Fig. 3).

In the following, we focus on the results of the multivariable regression models. The TUG performance was positively associated (i.e., it was negatively associated with the TUG time) with, the maximum distance from home, the convex hull area, the perimeter of the convex hull area, and the standard ellipse area. The TUG performance was borderline positively associated with the LSA and the daily distance covered during the week. We did not find evidence for an association between any life-space measure and the NIHSS or mRS (Table 3, Fig. 2, and Figures in the appendix).

**Table 3:** Outcomes of multivariable regression analyses for the six GNSS-based life-space outcomes and the LSA

Independent variable	LSA [0–120, higher is better]		Total covered distance [m]^a^		Maximum distance from home [m]^a^	
	Estimate	*p*-value	Estimate	*p*-value	Estimate	*p*-value
Timed up-and-go time [s]^a^	– 33.64 [– 68.02; 0.74]	0.055	– 1.07 [– 2.21; 0.07]	0.064	– 2.62 [– 4.43; – 0.81]	0.006
NIHSS (≥ 2 over < 2)	13.14 [– 6.98; 33.27]	0.193	– 0.10 [– 0.77; 0.56]	0.756	0.05 [– 1.01; 1.11]	0.928
mRankin (≥ 2 over < 2)	2.72 [– 17.05; 22.50]	0.781	0.09 [– 0.56; 0.75]	0.777	0.09 [– 0.95; 1.1]	0.860

Independent variable	Convex hull area [km^2^]^a^		Perimeter of the convex hull area [km]^a^		Standard ellipse area [km^2^]^a^	
	Estimate	*p*-value	Estimate	*p*-value	Estimate	*p*-value
Timed up-and-go time [s]^a^	– 4.28 [– 7.41; – 1.15]	0.009	– 2.26 [– 3.90; – 0.62]	0.008	– 4.42 [– 8.19; – 0.66]	0.023
NIHSS (≥ 2 over < 2)	– 0.13 [– 1.96; 1.70]	0.887	0.07 [– 0.89; 1.03]	0.884	0.05 [– 2.16; 2.25]	0.967
mRankin (≥ 2 over < 2)	– 0.02 [– 1.82; 1.78]	0.982	0.07 [– 0.87; 1.02]	0.877	– 0.51 [– 2.68; 1.66]	0.636

All models were adjusted for age, sex, and residential area

^a^Log-transformed data

## Discussion

### Main findings

We identified positive associations between several distance- and area-related life-space measures and the TUG performance and a borderline significant association between the LSA score and the TUG performance. We did not observe a statistically significant association of any life-space measure with the NIHSS or mRS.

### Interpretation of results

The observed higher values in life space (i.e., objectively measured maximal distance from home and area-based measures) in participants with a better physical function (i.e., better TUG performance) appear to be reasonable based on findings reported for questionnaire-based life-space assessments in previous studies in patients with stroke [20, 22, 42, 47]. The ability to get up from a chair and walk is an essential component of being able to move within the life space. Physical function could be seen as a pre-requisite for the resulting life space.

### Comparison with other studies

Ho et al. (2021) compared 66 people with stroke (mean age 64.9 ± 6.6 years) with 65 healthy older people (mean age 67.0 ± 7.0 years) [20]. They assessed life space using LSA and compared the values with that of healthy older people with and without depressive symptoms. They reported a significant correlation of the LSA composite score with the Fugl-Meyer Assessment of lower extremities score (*r*s = 0.31), the Five Times Sit-To-Stand time (*r*s = − 0.43), and the Frenchay Activities Index score (*r*s = 0.48) and concluded that patients with stroke had significantly lower LSA composite scores than healthy older people [20]. We found a borderline significant association between the LSA score and the TUG performance in our study and the GNSS-based life space showed a positive association with the TUG performance. As such, our findings are in line with the results of the above study.

In a longitudinal study, Tsunoda et al. (2021) investigated changes in life space in 89 community-dwelling chronic, stable patients after a stroke (median age 74 (67–79) years, 77% male) and analyzed which factors contributed to a change in life space [42]. They assessed life space with the LSA at three-time points (at baseline, as well as 12 and 24 months post stroke). The authors used comfortable gait speed, the Functional Independence Measure Motor subscale (FIM motor) and the Mini-Mental State Examination (MMSE) to assess physical function, functional independence, and cognition. The multivariable linear mixed-effects model showed that the LSA scores declined significantly over the two-year period. Further, the authors found that the comfortable gait speed and age were significantly associated with changes in the LSA score, independent of FIM motor scores and MMSE scores. They concluded that gait speed is a predominant factor affecting life space and that, therefore, regular assessment of gait function and appropriate strategies are needed to prevent deterioration of gait speed in chronic post-stroke patients [42]. The authors reported higher life-space values in patients with higher physical function which is in line with the findings of our study [42].

In a cross-sectional study, Yang et al. (2017) investigated life space in 34 patients with stroke (mean age 65.1 ± 2.4 years, 59% male) using the Korean version of the Life-Space Assessment (K-LSA) and the relationships between the K-LSA and physical function, daily activity, quality of life, and post-stroke depression [47]. In line with our findings, Yang et al. (2017) concluded that life space of post-stroke patients is associated with patients' functional mobility [47].

### Strengths and limitations

Our study has several strengths: it is one of few studies that objectively measured life space in patients with stroke and, to the best of our knowledge, the first study investigating life space of patients with stroke using both the LSA questionnaire and a GNSS-based assessment. We performed our study 3 months after the stroke, a time point commonly used to evaluate stroke outcome. Our cohort of ambulatory patients with stroke was treated at a comprehensive stroke care center, integrating acute stroke treatment at the stroke center of the University Hospital Basel and the Neurorehabilitation Center, Felix Platter, within the Stroke Treatment Chain Basel.

We chose to apply the GNSS-based assessment during a 1-week period following the on-site appointment. We defined a valid weekly assessment when the patient had one valid day with 6 h of GNSS recordings. This might be considered relatively short, however, there are studies that used a 7-day range before [19, 26] as well as other studies using only 3 or 5 days of GNSS measurement [4, 43].

There are some limitations that need to be discussed: there was a relatively high amount of missing GNSS data (17 out of 58, equaling 29%) which were due to technical errors of the device or user error. In the study of Giannouli et al. (2018), a comparably high dropout rate due to incomplete GPS data has been reported [15]. Previous studies reported similar problems related to the battery runtime of portable GNSS devices [37].

A new generation of tracking devices could overcome the major limitations that led to a relatively high amount of (partially) missing data in our study. Ideally, devices should allow longer battery run times and increased portability, preferably in a waterproof, wrist worn, watch-shaped housing. Such devices could highly improve compliance and allow for a more reliable life-space assessment, leading to improved quality and completeness of data.

We did not observe differences regarding the participant characteristics between the two groups with and without GNSS. The majority of these errors could be avoided in the future with a lower burden for the participants (e.g. longer battery runtime without the need of charging the device every night during the measurement period; smaller, waterproof devices, possibly worn at the wrist). Our study is a cross-sectional study, therefore not allowing the analysis and interpretation of the evolvement of life-space performance following stroke.

The measurements took place between January 2020 and February 2022. Research suggests that the observed life space could have generally been reduced due to COVID-19 measures compared to pre-pandemic levels [35]. Anonymized data from mobile phone usage in Italy, Portugal, and Spain suggest that mobility restrictions due to COVID-19 were smaller in older people (i.e. above 65 years of age) compared to younger people [9]. The latter finding is in contrast to other research reporting that particularly older people and vulnerable people were affected by COVID-19-related mobility restrictions [12, 31, 40]. The specific circumstances during the COVID-19 pandemic should be considered when interpreting the results of the study at hand.

## Conclusion

Our study showed that the easy-to-assess TUG performance is positively associated with life space 3 months after stroke. This highlights the importance of measuring and improving functional mobility to achieve meaningful recovery during rehabilitation post stroke.

Given the large variance in the life-space data that we observed in our study, future studies should aim for larger sample sizes to have higher statistical power to perform hypothesis testing.

## Supplementary Information

Below is the link to the electronic supplementary material.

Appendix 1: Visualization of the regression outcomes showing the unstandardized regression coefficients (B) and 95%-CIs of the models for the log-transformed total covered distance. The vertical line indicates the null value. Abbreviations: mRS (Modified Rankin Scale), NIHSS (National Institutes of Health Stroke Scale), TUG (timed up-and-go test)

Appendix 2: Visualization of the regression outcomes showing the unstandardized regression coefficients (B) and 95%-CIs of the models for the log-transformed maximum distance from home. The vertical line indicates the null value. Abbreviations: mRS (Modified Rankin Scale), NIHSS (National Institutes of Health Stroke Scale), TUG (timed up-and-go test)

Appendix 3: Visualization of the regression outcomes showing the unstandardized regression coefficients (B) and 95%-CIs of the models for the log-transformed perimeter of the convex hull area. The vertical line indicates the null value. Abbreviations: mRS (Modified Rankin Scale), NIHSS (National Institutes of Health Stroke Scale), TUG (timed up-and-go test)

Appendix 4: Visualization of the regression outcomes showing the unstandardized regression coefficients (B) and 95%-CIs of the models for the log-transformed standard ellipse area. The vertical line indicates the null value. Abbreviations: mRS (Modified Rankin Scale), NIHSS (National Institutes of Health Stroke Scale), TUG (timed up-and-go test)

## Data Availability

The data that support the findings of this study are available from the corresponding author upon reasonable request.

## Abbreviations

BMI: Body Mass Index
GALILEO: Global navigation satellite system by the European Union through the European Space Agency
GDS-15: Geriatric Depression Scale
GLONASS: Globalnaja nawigazionnaja sputnikowaja sistema
GNSS: Global Navigation Satellite System
GPS: Global Positioning System
IADL: Instrumental Activities of Daily Living
LSA: University of Alabama at Birmingham Study of Aging Life-Space Assessment
MOBITEC-Stroke: Recovery of mobility function and life-space mobility after ischemic stroke
MoCA: Montreal Cognitive Assessment
mRS: Modified Rankin Scale
NIHSS: National Institutes of Health Stroke Scale
TUG: Timed Up-and-Go test
