# Supplementary figures and images for: Timed up-and-go performance is associated with objectively measured life space in patients 3 months after ischemic stroke: a cross-sectional observational study
Source: J Neurol. 2022 Dec 22;270(4):1999–2009. doi: 10.1007/s00415-022-11524-x (PMC9772599; doi:10.1007/s00415-022-11524-x)

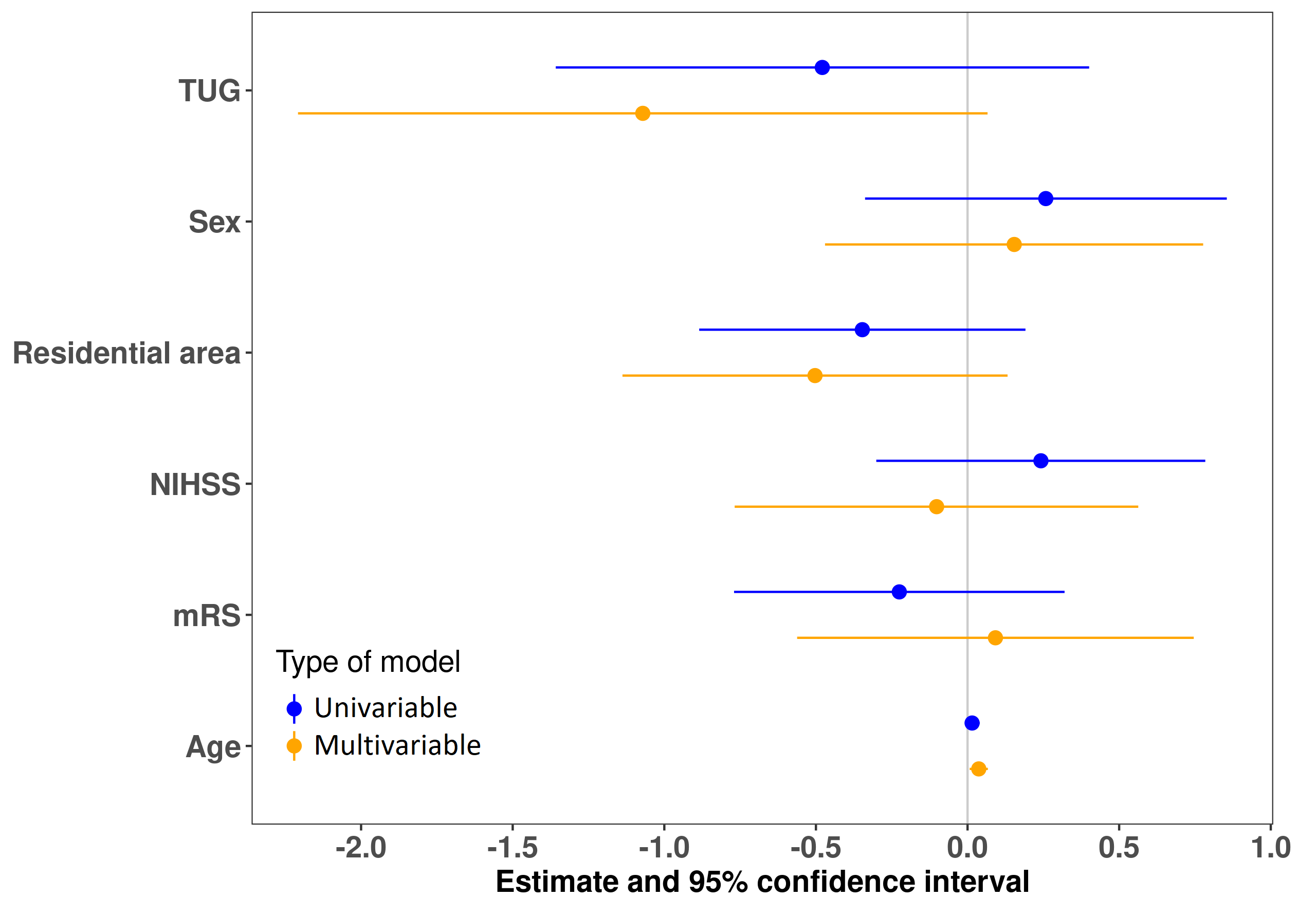

Supplement: Supplementary file 1 — Appendix 1: Visualization of the regression outcomes showing the unstandardized regression coefficients (B) and 95%-CIs of the models for the log-transformed total covered distance. The vertical line indicates the null value. Abbreviations: mRS (Modified Rankin Scale), NIHSS (National Institutes of Health Stroke Scale), TUG (timed up-and-go test) [file 415_2022_11524_MOESM1_ESM.tif]

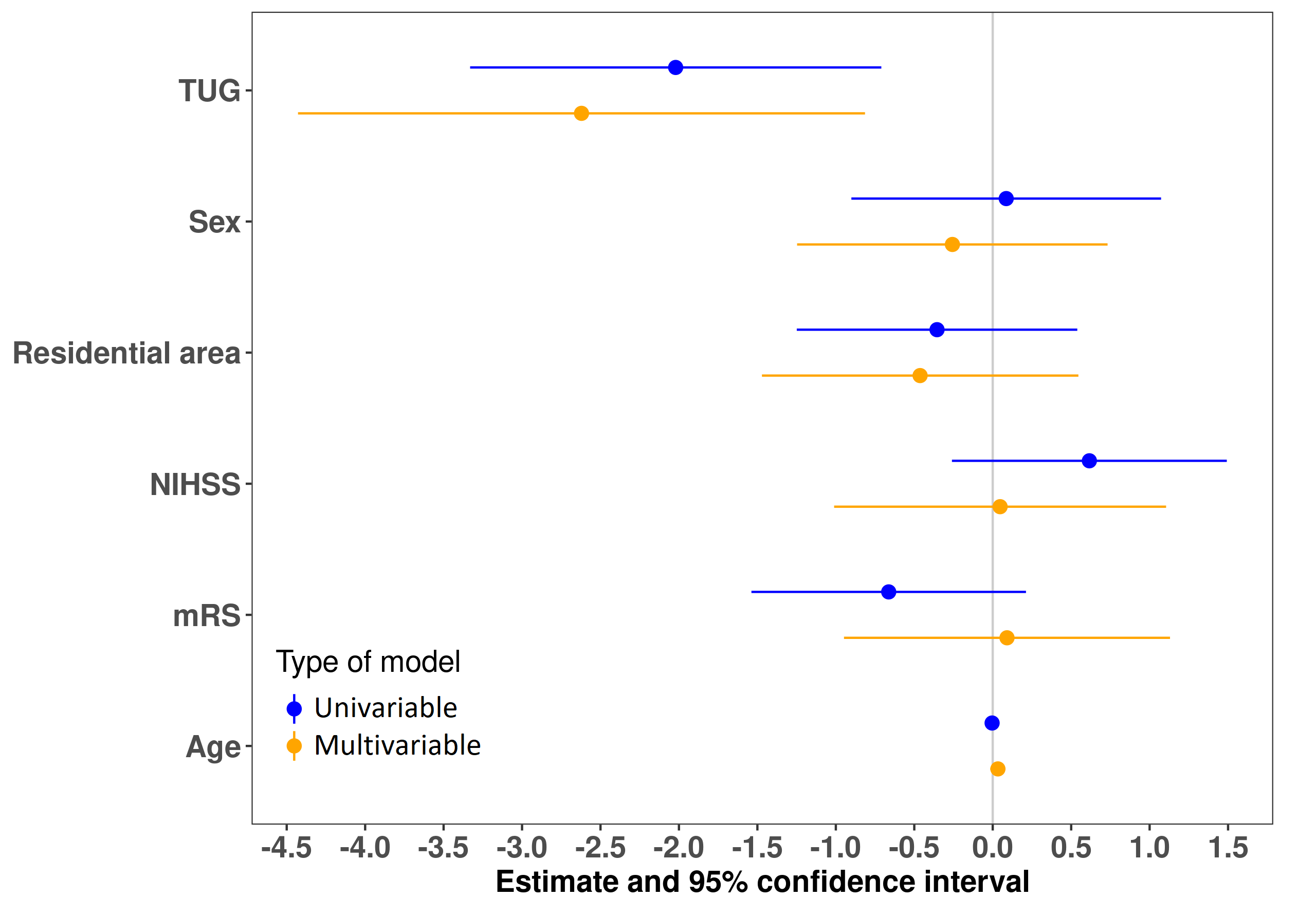

Supplement: Supplementary file 2 — Appendix 2: Visualization of the regression outcomes showing the unstandardized regression coefficients (B) and 95%-CIs of the models for the log-transformed maximum distance from home. The vertical line indicates the null value. Abbreviations: mRS (Modified Rankin Scale), NIHSS (National Institutes of Health Stroke Scale), TUG (timed up-and-go test) [file 415_2022_11524_MOESM2_ESM.tif]

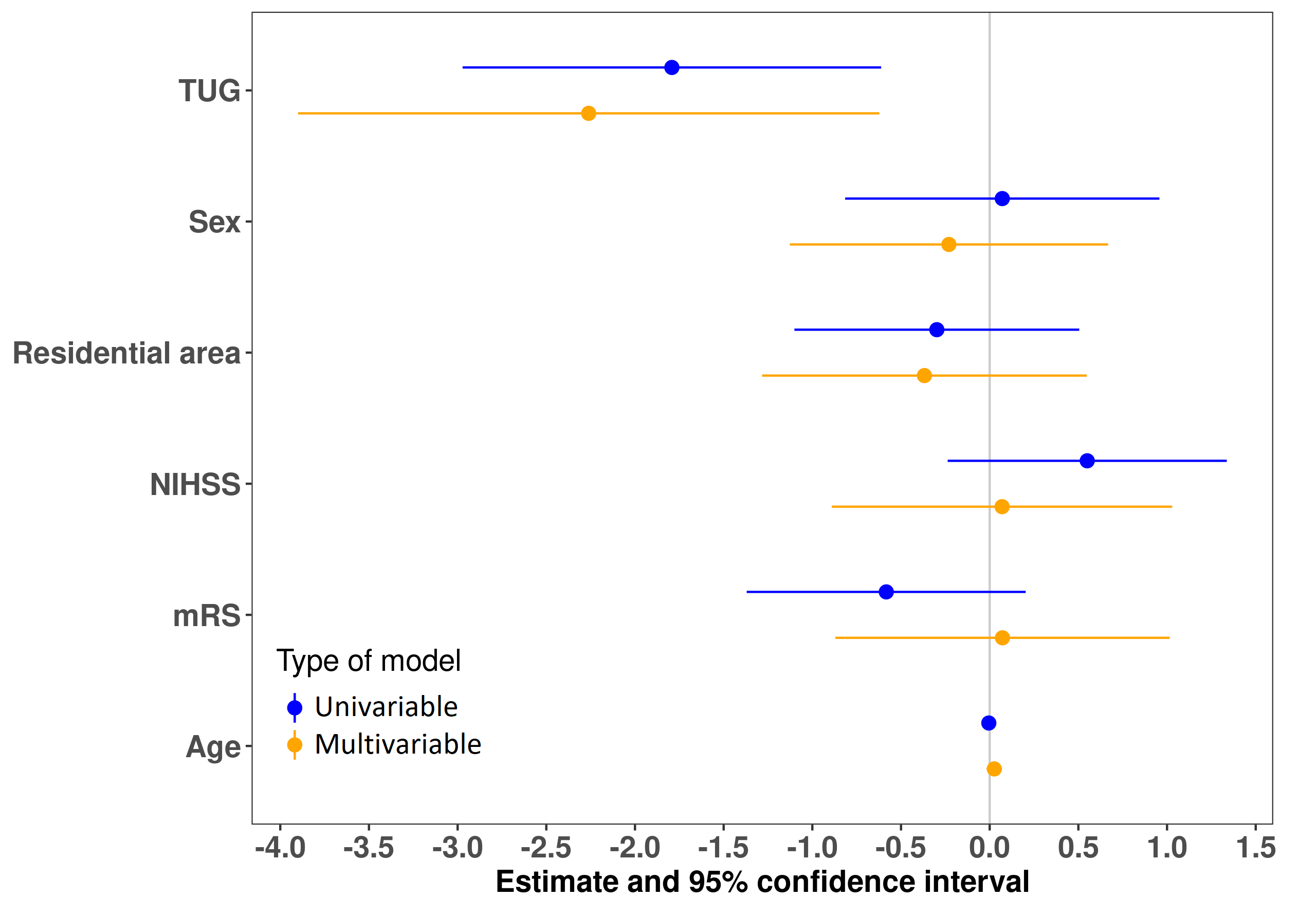

Supplement: Supplementary file 3 — Appendix 3: Visualization of the regression outcomes showing the unstandardized regression coefficients (B) and 95%-CIs of the models for the log-transformed perimeter of the convex hull area. The vertical line indicates the null value. Abbreviations: mRS (Modified Rankin Scale), NIHSS (National Institutes of Health Stroke Scale), TUG (timed up-and-go test) [file 415_2022_11524_MOESM3_ESM.tif]

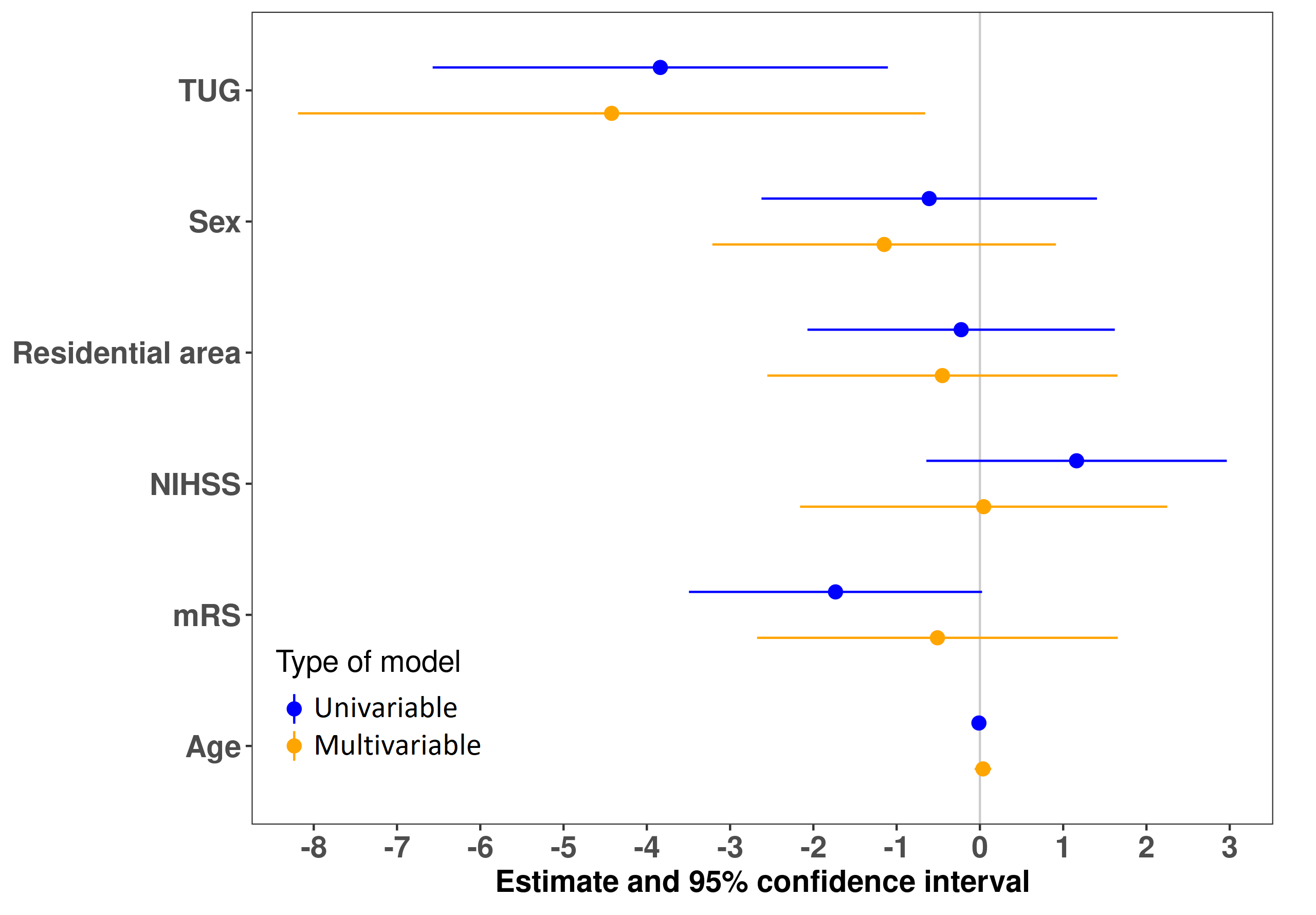

Supplement: Supplementary file 4 — Appendix 4: Visualization of the regression outcomes showing the unstandardized regression coefficients (B) and 95%-CIs of the models for the log-transformed standard ellipse area. The vertical line indicates the null value. Abbreviations: mRS (Modified Rankin Scale), NIHSS (National Institutes of Health Stroke Scale), TUG (timed up-and-go test) [file 415_2022_11524_MOESM4_ESM.tif]
